# Exosomes derived from miR‐181‐5p‐modified adipose‐derived mesenchymal stem cells prevent liver fibrosis *via* autophagy activation

**DOI:** 10.1111/jcmm.13170

**Published:** 2017-04-06

**Authors:** Ying Qu, Qidi Zhang, Xiaobo Cai, Fei Li, Zhenzeng Ma, Mingyi Xu, Lungen Lu

**Affiliations:** ^1^ Department of Gastroenterology & Hepatology Shanghai General Hospital Shanghai Jiao Tong University School of Medicine Shanghai China

**Keywords:** liver fibrosis, ADSC, exosomes, miRNA, autophagy

## Abstract

Proliferating hepatic stellate cells (HSCs) respond to liver damage by secreting collagens that form fibrous scar tissue, which can lead to cirrhosis if in appropriately regulated. Advancement of microRNA (miRNA) hepatic therapies has been hampered by difficulties in delivering miRNA to damaged tissue. However, exosomes secreted by adipose‐derived mesenchymal stem cells (ADSCs) can be exploited to deliver miRNAs to HSCs. ADSCs were engineered to overexpress miRNA‐181‐5p (miR‐181‐5p‐ADSCs) to selectively home exosomes to mouse hepatic stellate (HST‐T6) cells or a CCl4‐induced liver fibrosis murine model and compared with non‐targeting control Caenorhabditis elegans miR‐67 (cel‐miR‐67)‐ADSCs. *In vitro* analysis confirmed that the transfer of miR‐181‐5p from miR‐181‐5p‐ADSCs occurred *via* secreted exosomal uptake. Exosomes were visualized in HST‐T6 cells using cyc3‐labelled pre‐miRNA‐transfected ADSCs with/without the exosomal inhibitor, GW4869. The effects of miRNA‐181‐5p overexpression on the fibrosis associated STAT3/Bcl‐2/Beclin 1 pathway and components of the extracellular matrix were assessed. Exosomes from miR181‐5p‐ADSCs down‐regulated Stat3 and Bcl‐2 and activated autophagy in the HST‐T6 cells. Furthermore, the up‐regulated expression of fibrotic genes in HST‐T6 cells induced by TGF‐β1 was repressed following the addition of isolated miR181‐5p‐ADSC exosomes compared with miR‐67‐ADSCexosomes. Exosome therapy attenuated liver injury and significantly down‐regulated collagen I, vimentin, α‐SMA and fibronectin in liver, compared with controls. Taken together, the effective anti‐fibrotic function of engineered ADSCs is able to selectively transfer miR‐181‐5p to damaged liver cells and will pave the way for the use of exosome‐ADSCs for therapeutic delivery of miRNA targeting liver disease.

## Introduction

Liver fibrosis results from the activation of hepatic stellate cells (HSCs) through regulatory processes involving cytokines and growth factors 1. Proliferating HSCs contribute to an accumulation of extracellular matrix by secreting collagens that form fibrous scar tissue, which is beneficial to initial tissue repair but can lead to cirrhosis if inappropriately regulated 2, 3. Transforming growth factor‐β1 (TGF‐β1) is known to be a principal profibrogenic cytokine in the liver 3. TGF‐β1 indirectly induces collagen I expression and α‐smooth muscle actin (α‐SMA) stress fibre organisation by initiating the activation of quiescent HSCs 4. Signal transducer and activator of transcription 3 (STAT3) are a main mediator of interleukin‐6‐type cytokine signalling and are believed to be involved in liver fibrosis, but its role is unclear 5, 6, 7. Recent reports indicate that STAT3 may up‐regulate TGF‐β1 8. However, Seo *et al*. 9 have found that phospholipase D1 (PLD1), which catalyses the hydrolysis of phosphatidylcholine to yield phosphatidic acid, can reduce the levels of collagen I associated with fibrosis through autophagy, and that this process is independent of TGF‐β1 signalling.

The ability to control the factors and pathways leading to fibrosis could lead to the development of therapeutic agents. Several recent developments in this area have involved the use of microRNAs (miRNAs) 10, 11, 12, 13. However, there are difficulties in delivering miRNA to CCl4‐induced liver fibrosis models. Exosomes are small (30–100 nm in diameter) extracellular membrane vesicles containing proteins, and nucleic acids that are secreted by various cell types including HSCs and mesenchymal stem cells (MSCs) 14. Their main function is in cell communication where they deliver cell‐type specific proteins and nucleic acids, including mRNAs and miRNAs, to regulate intracellular signalling pathways 15. Injured epithelial cells have been found to produce an increased number of exosomes with the genetic information required to activate fibroblasts 16. Borges *et al*. 16 discovered that exosomes released by injured epithelial cells promote proliferation, α‐SMA expression, F‐actin expression and type I collagen production in fibroblasts, and that fibroblast activation was dependent on the delivery of TGF‐β1 mRNA in exosomes. They proposed that TGF‐β1 was part of a rapid response to tissue repair and regeneration.

The miR‐181 family of miRNAs have been associated with maintaining an undifferentiated state of hepatic progenitor cells 17. They have also been implicated in regulating autophagy 18. Furthermore, STAT3 and Bcl‐2 have potential 3′‐UTR binding sites for miR‐181‐5p. In this work, we exploit the ability of exosomes to deliver nucleic acids to cells by up‐regulating miR‐181‐5p in adipose‐derived mesenchymal cells (ADSCs) selectively homed to HST‐T6 or a CCl4‐induced liver fibrosis murine model. We investigate the roles of STAT3/Bcl‐2 and TGF‐β1 signalling in fibrosis and the influence of miR‐181‐5p in mediating autophagy to counteract the accumulation of extracellular matrix components.

## Methods

### Cell treatments and transfections

Exosomes were isolated from conditioned medium of serum‐starved passage 5–10 primary mouse HSCs as described previously 19. Adipose tissue was collected from 10 normal C57BL/6 mice, aged 4–16 weeks and weighing 20–25 g, by the following method 20. Briefly, adipose tissue was isolated from regions close to the ovarian infundibulum in females and the lateral epididymis in males. Small pieces were incubated with type IV collagenase 0.2 U/ml for 3 hrs at 37°C in 5% CO_2_ then neutralized with 10 ml foetal bovine serum (Sigma‐Aldrich, St. Louis, MO, USA). After removing cellular debris, the vascular cells, fibroblasts and adipocytes were solubilized in Hank's balanced salt solution, filtered through a 40 μm membrane and centrifuged for 10 min at 274 × *g*, 4°C. The cell pellet was resuspended in 1 ml of complete culture medium and grown to subconfluence. Cells were resuspended in culture medium, trypsinized and filtered. The filtrate was collected in cell culture flasks containing 50 ml of saline solution supplemented with antibiotics.

### Isolation and identification of ADSCs

Adipose tissue‐derived mesenchymal stem cells (ADSCs) became visible as large flat cells in the primary culture after three passages. Fluorescence activated cell sorting analysis of the isolated ADSCs was performed using a BD LSRII analyzer (BD bioscience, New Jersey). Spontaneous differentiation was not observed during culture expansion. Osteogenic differentiation was induced by culturing ADSCs for 21 days in Dulbecco's modified Eagle's medium (DMEM) supplemented with 10% foetal bovine serum (FBS), 0.1 μM dexamethasone, 50 μM ascorbate‐2‐phosphate and 10 mM beta‐glycerophosphate. Adipogenic differentiation was induced by culturing ADSCs for 14 days in DMEM supplemented with 10% FBS, 0.5 mM isobutylmethylxanthine, 1 μM dexamethasone, 10 μM insulin and 200 μM indomethacin. The ADSCs were observed to have undergone either osteogenic or adipogenic differentiation by staining with Alizarin Red S and Oil‐Red O, respectively.

### Exosome extraction

Adipose tissue‐derived mesenchymal stem cells (ADSCs) were either transfected with a plasmid encoding miR‐181‐5p or cel‐miR‐67 (Caenorhabditis elegans miR‐67) as a control by the following method 21. Prior to transfection, 1 × 10^6^ ADSCs were seeded in 10 ml of ADSC‐conditioned medium overnight. ADSCs were then transfected with plasmids encoding miR‐181‐5p or cel‐miR‐67 using Lipofectamine 2000 (Invitrogen, Carlsbad, CA, USA). After 48 hrs of miRNA transfection, exosomes were isolated from the ADSCs supernatant using an ExoQuick‐TC Kit (System Biosciences, CA, USA) according to manufacturer's instructions. The protein content of the exosomes was measured using a BCA™ Protein Assay Kit (Beyotime, Nantong, China). Exosomes were then analysed by Western blotting or visualized in HST‐T6 cells using cyc3‐labelled pre‐miRNA‐transfected ADSCs with/without the exosomal inhibitor, GW4869 (10 M for 48 hrs).

### Transmission electron microscopy

Exosomes obtained by the above method were precipitated from medium with ExoQuick TC (System Biosciences) using the manufacturer's instruction. Exosome pellets (~5 μl) were transferred to carbon‐coated 200‐mesh copper electron microscopy grids and incubated at room temperature for 5 min. The exosomes were then stained with uranyl acetate and washed three times in PBS. After being left to dry at room temperature, they were observed under a transmission electron microscope (Hitachi H7500 TEM, Tokyo, Japan). Micrographs were used to identify and quantify exosomes in ADSCs.

### Animals

All experiment protocols were approved by the ethics committee on animal use at our institute. Six‐week‐old male C57BL/6 mice were acclimatized for 2 weeks before use; mice were 8 weeks old at the time of the experiments. The animals were cared for in accordance with protocols approved by the Animal Care and Use Committee of Shanghai Jiao Tong University School of Medicine.

### Induced liver injury by CCl4 and exosome administration

A total of 32 mice were randomly divided into four groups (*n* = 8). A single dose of CCl4 (3% vol/vol in olive oil) was delivered to mice at 0.05 ml/kg body weight by intraperitoneal injection twice each week for 8 weeks. Sham mice were administered with the same volume of olive oil only for 8 weeks. Mice were concomitantly treated with CCl4 with or without exosomes for 8 weeks. Exosomes (0.4 μg/μl, 100 μl) were administered through intrasplenic injection twice each week for 8 weeks. The control group received 100 μl PBS. Mice were killed 24 hrs after CCl4. Livers and serum were collected for further analysis.

### Cytokine assay and serum measurements

Enzyme‐linked immunoassays (ELISAs) were used to detect cytokines in the liver tissue collected above. ELISA kits for TNF‐α, IL‐6 and IL‐17 were purchased from Boster system (Boster, China), and the assays were performed according to manufacturer's instructions. To assess hepatic damage and function, levels of aspartate aminotransferase (AST), alanine aminotransferase (ALT) and total bilirubin were measured in serum using commercial kits and a semiautomated photometer 5010 (Robert Riele GmbH & Co KG, Berlin, Germany).

### Western blot analysis

Proteins extracted from cell pellets were loaded on to 12% sodium dodecyl sulphate‐polyacrylamide gel electrophoresis (SDS‐PAGE) and then transferred onto polyvinylidene fluoride (PVDF) membranes (Millipore, Bedford, MA, USA). The membranes were incubated first with primary antibody and then with secondary antibody. GAPDH was used as a control to verify the equal loading of proteins. Western blot analysis was carried out by a standard protocol using antibodies for STAT3, Bcl‐2, Beclin 1, LC3 and p62 purchased  from Cell Signalling Technology, Inc. (Beverly, MA, USA); antibodies for α‐SMA were from Dako (Glostrup, Denmark); and antibodies for collagen I, fibronectin and GAPDH were from Santa Cruz Biotechnology (Santa Cruz, CA, USA).

### Real‐time RT‐PCR analysis

RT‐PCR analysis was performed on a [RT‐PCR machine]. All reactions were run in triplicate. The cycle threshold (Ct) method was used to calculate values. Ct values were normalized to the GAPDH gene as a cDNA loading control, and changes were calculated relative to controls. Total RNA was extracted from cells using the TRIzol reagent (Invitrogen, Carlsbad, CA), followed by real‐time PCR analysis with ABI Prism 7900 (Applied Biosystems, Foster City, CA, USA) to examine the expression of Collagen I, Collagen III and Fibronectin in cells and TNFα, IL‐6 and IL‐17 in live tissue.

### Transfections and dual‐luciferase assay

HST‐T6 cells were transfected with firefly luciferase transcript containing either wild‐type or mutant form of 3′‐UTR of the two candidate genes, in the presence of either control or miR‐181‐5p, and then assessed for luciferase reporter activity at 24 hrs post‐transfection. Harvested cells were used in a dual‐luciferase reporter assay system (Promega, Madison, WI, USA) for the sequential measurement of firefly and *Renilla* luciferase activities with specific substrates. A luminometer (TD‐20/20; Turner Designs, Sunnyvale, CA, USA) was used to quantify luciferase activities and to calculate the relative ratios.

### LC3 puncta formation

To monitor the formation of light chain‐3 (LC3) puncta, cells incubated with exosomes for up to 24 hrs. Then cells were transiently transfected with red fluorescent protein (RFP)‐LC3 and then cultured under nutrient starvation conditions such as on HBSS (Hank's Buffered Salt Solution; amino acid‐free) medium. The cells were then fixed with 4% paraformaldehyde for fluorescence microscopy and visualized, and the images were collected using a fluorescence microscope (Axiovert200 M, Zeiss, Wetzlar, Germany).

### Immunofluorescence staining

TGF‐β‐induced HSC‐T6 cells were treated with miR‐181‐5p exosomes (Exo‐181) or cel‐miR‐67 (Exo‐67), then vimentin and α‐SMA were analysed by immunofluorescence. Vimentin and α‐SMA antibodies were purchased from Santa Cruz Biotechnology.

### Histological examination and immunohistochemistry

Mice liver tissues were stained with haematoxylin and eosin (H&E) and immunohistochemistry dye and observed at ×200 magnification. The liver sections were stained with haematoxylin and eosin (H&E) for histopathological examination. Immunohistochemical examinations were performed to detect the expression of Collagen I or Vimentin. In brief, the paraffin sections were deparaffinized and rehydrated. The sections were exposed to fresh 3% hydrogen peroxide for 20 min, and then washed with PBS. Antigens were retrieved in 0.01 M citric acid. The samples were incubated for 30 min at room temperature in 5% normal blocking serum, and incubated with Collagen I (Santa Cruz Biotechnology) or Vimentin overnight at 4°C. The slides were then incubated with secondary antibody for 60 min at room temperature, and with 3,3′‐diaminobenzidine as a substrate. Finally, the sections were counterstained with haematoxylin, and mounted.

### Statistical analysis

Data are expressed as mean ± SE. Two‐way ANOVA was applied to interpret the differences between treatment groups. Differences with a *P* value <0.05 were considered statistically significant.

## Results

### Exosome‐mediated miR‐181‐5p communication between ADSCs and HST‐T6 cells

**Figure 1:**
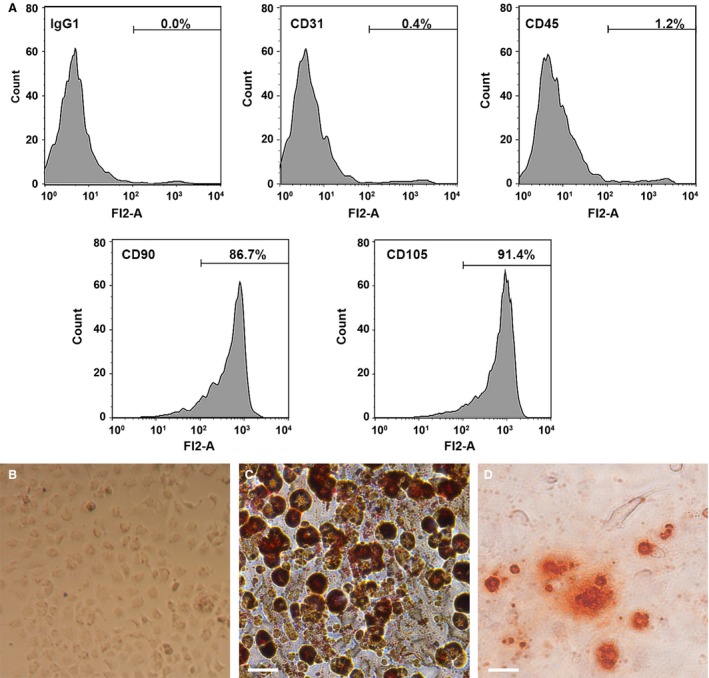
Identification of human adipose‐derived mesenchymal stem cells (ADSCs). (**A**) Flow cytometry analysis of the surface markers in ADSCs. (**B**) Cellular morphology of ADSCs in culture. (**C**) Oil Red O staining in ADSCs cultured in adipogenesis differentiation medium for 14 days. (**D**) Alizarin Red S staining in ADSCs cultured in osteogenesis differentiation medium for 21 days. Scale bar = 50 μm.

Flow cytometry analysis with cell surface specific markers was used to identify ADSCs (Fig. 1A). ADSCs were able to express CD90 and CD105 but were negative for, CD31 and CD45. Cellular morphology of ADSCs in culture is shown in Figure 1B. ADSCs were able to undergo multi‐lineage differentiation when grown in specific‐differentiation media. Adipogenesis of ADSCs was observed by Oil‐Red O staining (Fig. 1C). However, Alizarin Red S staining in ADSCs cultured in osteogenesis differentiation medium shows the mineralisation of the extracellular matrix, which confirms that osteogenesis has taken place (Fig. 1D).

**Figure 2:**
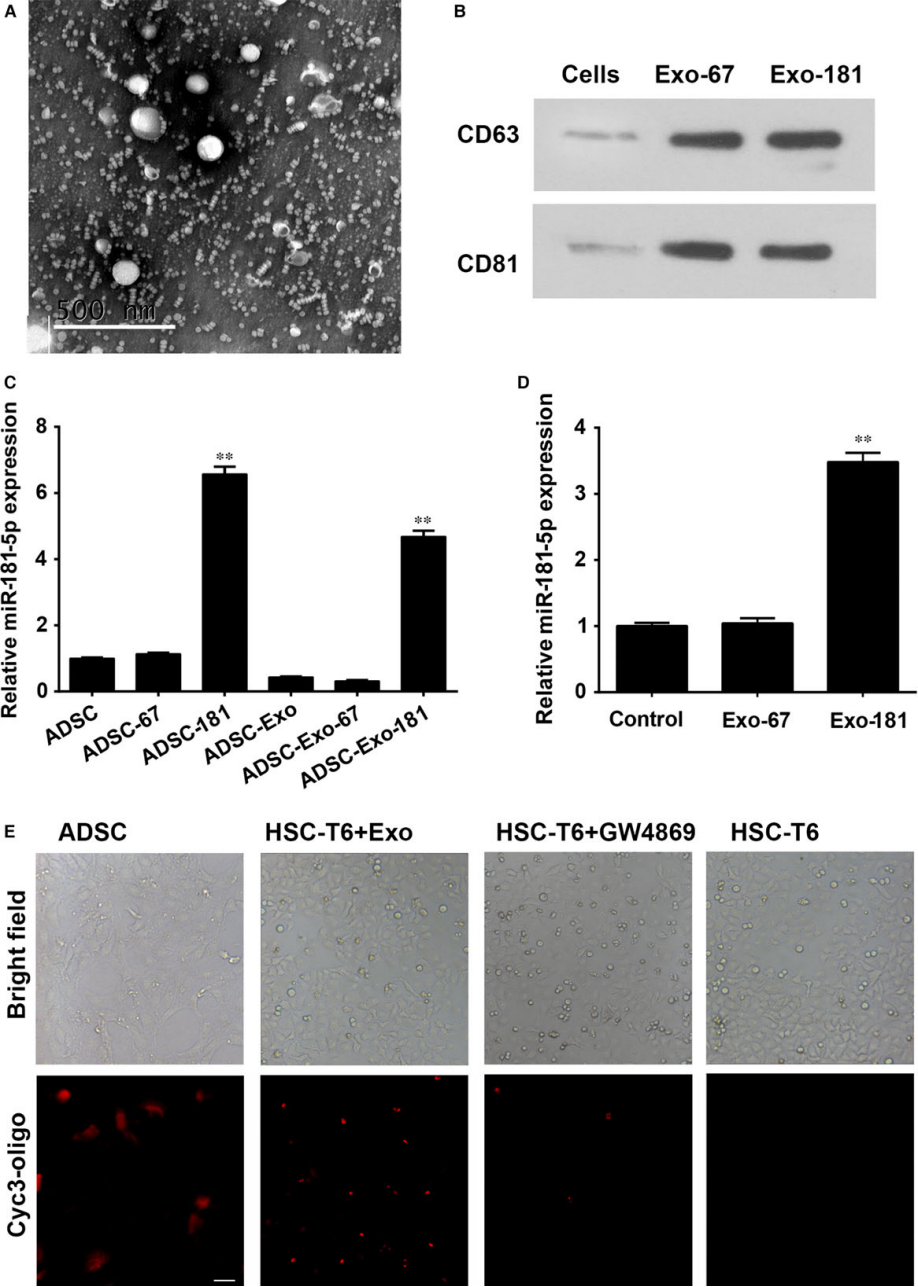
Exosome‐mediated miR‐181‐5p communication between adipose‐derived mesenchymal stem cells (ADSCs) and HST‐T6 cells. (**A**) Exosomes extracted from ADSCs were identified by TEM. Magnification: ×150,000. Scale bar: 500 nm (**B**): Western blot for CD63 and CD81 expression in ADSC‐derived exosomes. Real‐time PCR detection of miR‐181‐5p expression in ADSCs, ADSC‐derived exosomes (**C**) and exosome‐treated HST‐T6 cells (**D**). (**E**) Confocal images of ADSC‐181 stained with cyc3‐oligo. Transfer of fluorescent exosomes from ADSC‐181 is apparent in HST‐T6 cell membranes and cytoplasm. Data are presented as means ± SE. (**P < 0.01, *n* = 3). ADSC‐181: miR‐181‐5p‐transfected ADSCs; HST‐T6 + Exo: ADSC‐derived exosomes transfer of miRNA from the ADSCs to the target cells; HST‐T6 + GW4869:GW4869 (10 M for 48 hrs) exosomal inhibitor, was confirmed to inhibit the elevated expression of miR‐181‐5p in HST‐T6 cells when cocultured with miR‐181‐5p‐ADSCs. HST‐T6: Brightfield and immunofluorescence images showing HST‐T6 cells without cyc3‐oligo transfection. Scale bar = 100 μm.

To investigate the extracellular communication between ADSCs and HST‐T6, we first extracted exosomes from ADSCs identified by TEM (Fig. 2A). The exosome protein markers CD63 and CD81 were identified by Western blotting in exosomes derived from cel‐miR‐67 and miR‐181‐5p transfected ADSCs (Fig. 2B). Expression of miR‐181‐5p was significantly increased in ADSCs and in miR‐181‐5p exosomes (*P* < 0.001; Fig. 2C) and in HSTs transferred with miR‐181‐5p exosomes (*P* < 0.001; Fig. 2D) compared to untransfected cells and the cel‐miR‐67 controls. *In vitro* analysis confirmed that the transfer of miR‐181‐5p from miR‐181‐5p‐ADSCs occurred *via* secreted exosomal uptake, visualized in HST‐T6 cells using cyc3‐labelled pre‐miRNA‐transfected ADSCs with/without the exosomal inhibitor, GW4869 (Fig. 2E).

### miR‐181‐5p suppresses HSCs activation through direct targeting of Bcl‐2 and STAT3

**Figure 3:**
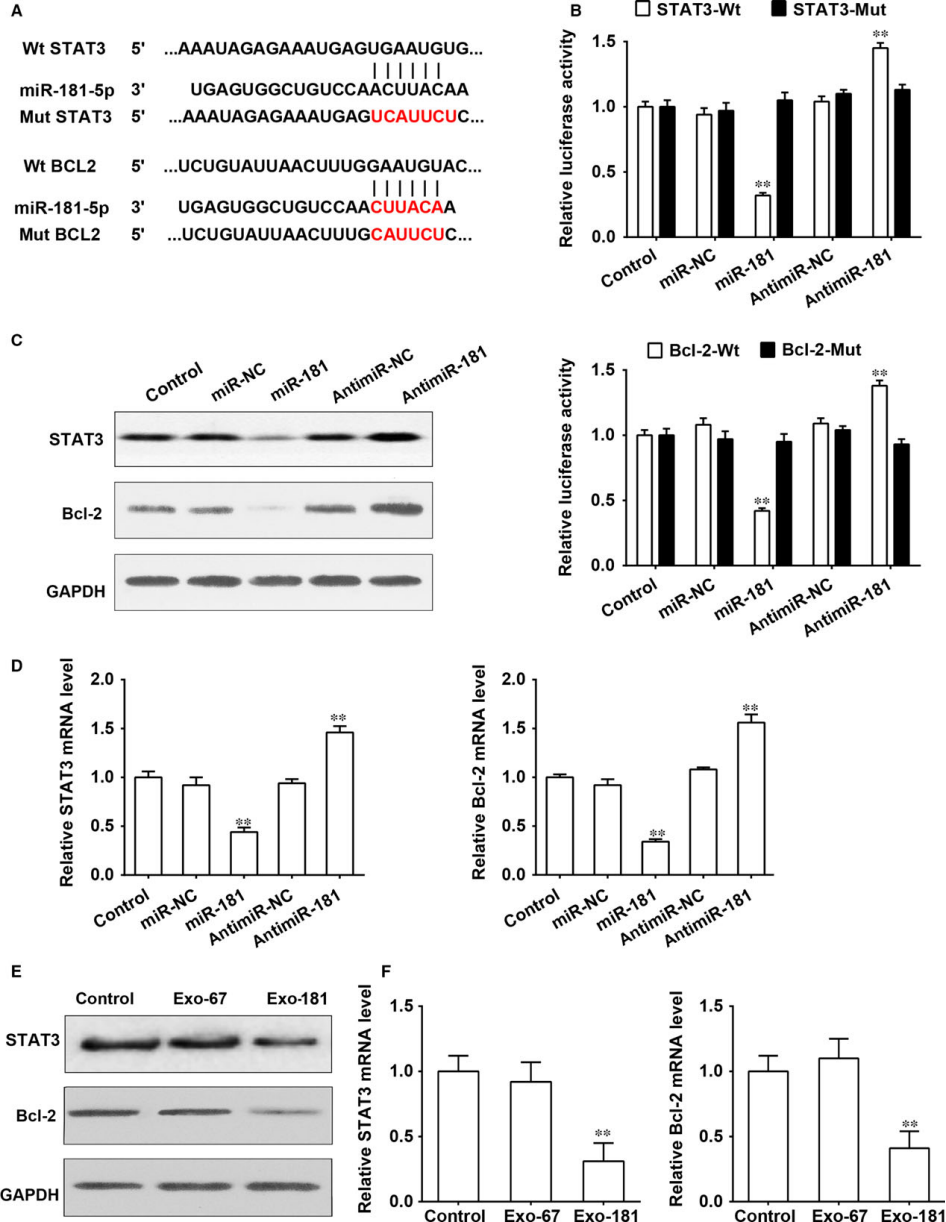
miR‐181‐5p suppresses HSCs activation through the direct targeting of Bcl‐2 and Stat3. (**A**) miR‐181‐5p potential binding sites on the 3′‐UTR of BCL‐2 and STAT3. (**B**) HST‐T6 cells were transfected with firefly luciferase transcript containing either a wild‐type or mutant form of 3′‐UTR of the two candidate genes, in the presence of either control or miR‐181‐5p, and then assessed for luciferase reporter activity at 48 hrs post‐transfection. The luciferase reporter activity of Bcl‐2 and Stat3 was suppressed by wild‐type miR‐181‐5p. (**C**) Protein expression of BCL‐2 and STAT3 was reduced by miR‐181‐5p in HST‐T6 cells. (**D**) mRNA expression of Bcl‐2 and Stat3 was reduced by miR‐181‐5p (**E**,** F**) Protein and mRNA expressions of BCL‐2 and STAT3 were reduced by exosomes derived from miR‐181‐5p‐modified ADSCs. **P < 0.01.

The potential binding sites of miR‐181‐5p on the 3′‐UTR of Bcl‐2 and Stat3 and the sites with point mutations inserted to prevent binding are shown in Figure 2A. Relative luciferase activity of HST‐T6 cells transfected with Bcl‐2 or Stat3 containing either mutated or wild‐type 3′‐UTR showed that the activity of BCL‐2 and STAT3 were suppressed by wild‐type miR‐181‐5p (*P* < 0.001, Fig. 3B). Increased luciferase activity in the presence of antimiR‐181‐5p confirmed the presence of functional miR‐181‐5p. The reduction in STAT3 and BCL‐2 expression was confirmed by western blotting (Fig. 3C) and by measuring mRNA levels (*P* < 0.001; Fig. 3D). Both protein and mRNA expression of BCL‐2 and STAT3 were reduced by exosomes derived from miR‐181‐5p‐modified ADSCs (*P* < 0.001; Fig. 2E and F).

### Exosome(miR‐181‐5p)‐induced autophagy is dependent on the STAT3/Bcl‐2/Beclin 1‐signalling pathway

**Figure 4:**
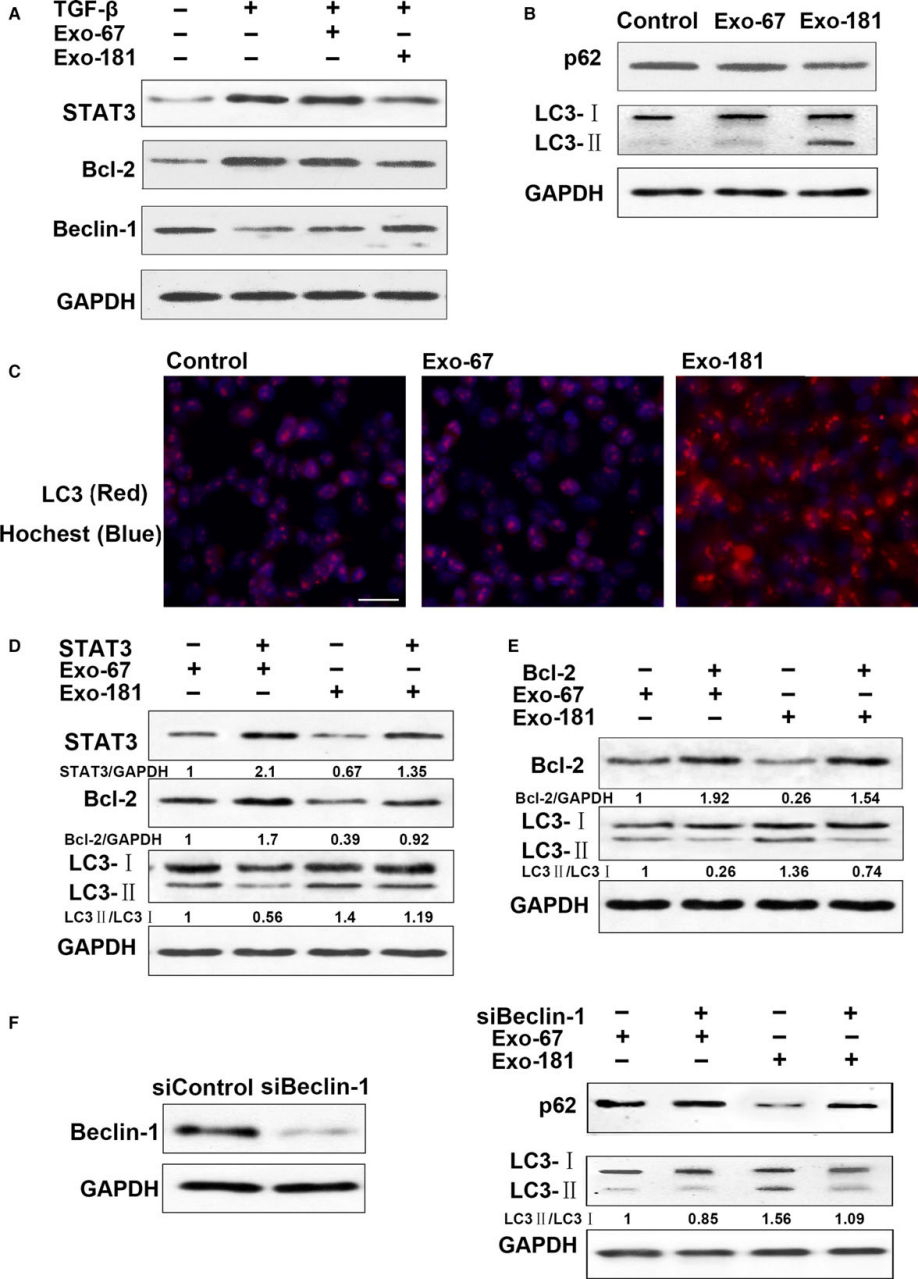
Exosomes of miR‐181‐5p (Exo‐181)‐induced autophagy through inhibition of the STAT3/Bcl‐2/Beclin 1‐dependent signalling pathway. (**A**) Exosomes reversed TGF‐β‐induced STAT3, Bcl‐2 up‐regulation and Beclin‐1 down‐regulation of expression in HSC‐T6 cells. HSCs were treated with TGF‐β at 2 ng/ml. (**B**) HSCs were treated with TGF‐β at 2 ng/ml. Exosomes‐induced autophagy assay by Western blot. (**C**) HSC‐T6 cells were infected with Exo‐181 for 24 hrs, and LC3 puncta formation was analysed by immunofluorescence. Scale bar = 100 μm (**D**): Effect of STAT3 on autophagy induced by Exo‐181 in HSC‐T6 cells. STAT3 up‐regulates Bcl‐2. Exo‐181 down‐regulates Bcl‐2. (**E**) Effect of Bcl‐2 on autophagy induced by Exo‐181 in HSC‐T6 cells. (**F**) Exo‐181‐induced autophagy dependent Beclin 1.

Western blots show that Exo‐181 reversed the up‐regulation of STAT3, up‐regulation of Bcl‐2 induced by TGF‐β and the down‐regulation of Beclin‐1 expression in HSC‐T6 cells where as Exo‐67 had no effect (Fig. 4A). Figure 3B and C represents the results of autophagy assays using the detection of LC3 puncta formation by Western blotting and immunofluorescence. In HSCs treated with TGF‐β1 at 2 ng/ml, Exo‐181 was found to induce autophagy (Fig. 4B), and these results were confirmed by immunofluorescence (Fig. 4C). STAT3 overexpression up‐regulates Bcl‐2 whereas miR‐181‐5p down‐regulates Bcl‐2 in HSC‐T6 cells (Fig. 4D). Bcl‐2 overexpression inhibits the autophagy induced by miR‐181‐5p in HSC‐T6 cells (Fig. 4E). Figure 3F shows that miR‐181‐5p‐induced autophagy is dependent on Beclin‐1 as autophagy is inhibited when Beclin‐1 is silenced.

### Exosomes(miR‐181‐5p) inhibit liver fibrosis *in vitro* and in CCl4‐induced liver fibrosis

**Figure 5:**
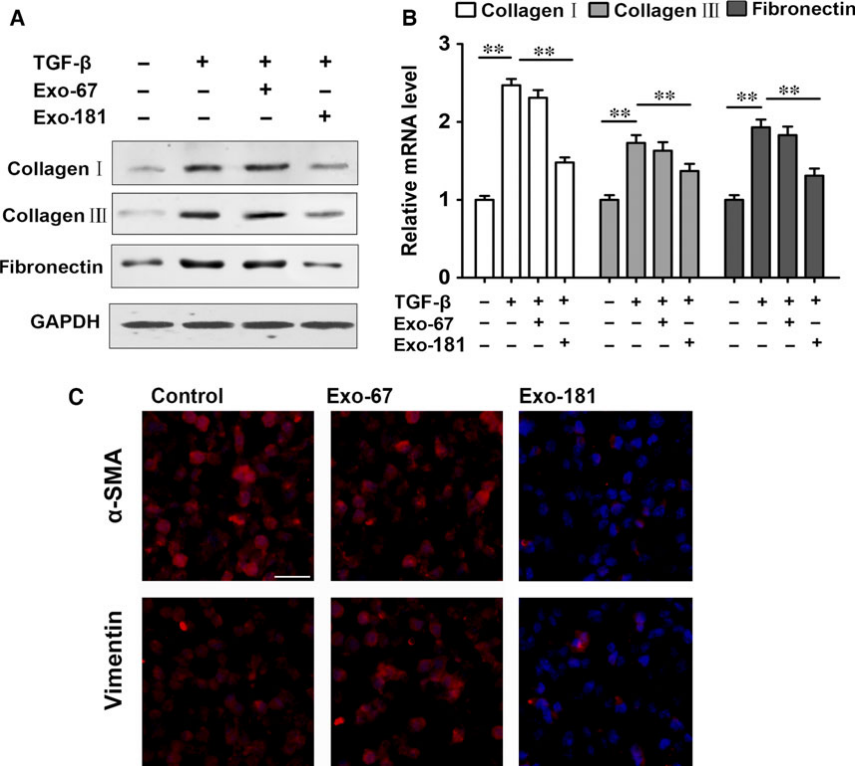
Exosomes(Exo‐181) inhibit liver fibrosis *in vitro*. Alteration of fibrosis‐related products induced by exosomes containing miR‐181‐5p (Exo‐181) in TGF‐β‐induced rat primary HSCs. (**A**) Western blot and (**B**) RT‐PCR for TGF‐β‐induced fibrosis‐related products and Exo‐181 reversed. (**C**) TGF‐β‐induced HSC‐T6 cells were infected with Exo‐181, and Vimentin and α‐SMA were analysed by immunofluorescence. Scale bar = 100 μm **P < 0.01.

**Figure 6:**
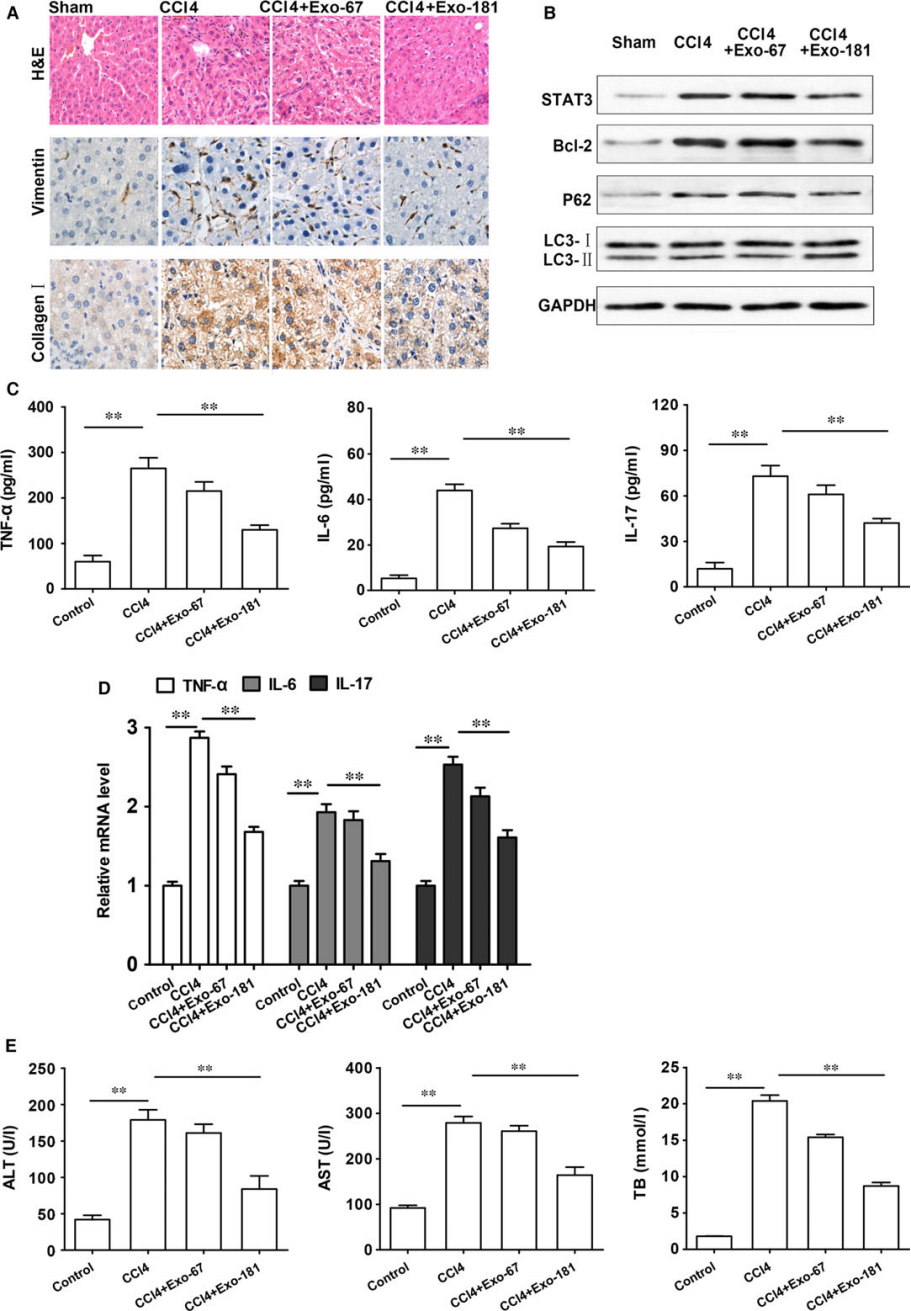
CCl4‐induced liver fibrosis is reduced by exosomes containing miR‐181‐5p (Exo‐181)‐induced autophagy compared to the control Exo‐67. (**A**) H&E and IF‐stained liver tissues taken from mice (×200 magnification). (**B**) Stat3 and Bcl‐2 expression and proteins of autophagy gene expression. (**C**) RT‐PCR assay and (D) ELISA for TNFα, IL‐6 and IL‐17 (**E**) Exosomes reduced liver‐injury level in ALT, AST and TB after CCl4 treatment. **P<0.01

The up‐regulation of fibrosis‐related products collagen I and fibronectin induced by TGF‐β in HSCs was reversed by Exo‐181 as shown by Western blots (Fig. 5A) and RT‐PCR (Fig. 4B). TGF‐β‐induced HSC‐T6 cells infected with Exo‐181 and analysed by immunofluorescence show a similar result for vimentin and α‐SMA (Fig. 5C). Figure 6A shows collagen I and vimentin levels in H&E and IF‐stained liver tissues taken from mice with CCl4‐induced liver fibrosis. Collagen I and vimentin levels are clearly reduced in mice injected with Exo‐181 compared with those injected with Exo‐67. Western blots show that STAT3 and Bcl‐2 expressionare up‐regulated in response to CCl4 treatment and that these levels are reduced in mice injected with Exo‐181 along with increases in P62 (Fig. 6B). Levels of LC3 puncta associated with autophagyare were also increased.

We next assessed the effects of exosome delivery of Exo‐181 on inflammatory factors by measuring the expression levels of TNFα, IL‐6 and IL‐17 by RT‐PCR and ELISA. There was a significant decrease in the protein levels of TNFα, IL‐6 and IL‐17 (*P* < 0.01) in CCI4 treated fibrosis‐induced mice injected with Exo‐181 compared to the liver fibrosis mouse model without exosome treatment (Fig. 6C). Levels of mRNA gave similar results (Fig. 6D). Exo‐181 injected mice also had reduced levels of liver injury as demonstrated by the significantly decreased serum levels of ALT, AST and TB after CCl4 treatment (*P* < 0.01; Fig. 6D).

## Discussion

Therapies using miRNA are a promising means of regulating the pathways involved in liver fibrosis. Here, we describe a process by which exosomes containing miRNA‐181‐5p can regulate autophagy in HST‐T6 cells and liver cells in mice to subsequently lower the levels of extracellular matrix components. *In vitro* analysis confirmed that the transfer of miR‐181‐5p from miR‐181‐5p‐ADSCs occurred *via* secreted exosomal uptake, visualized in HST‐T6 cells using cyc3‐labelled pre‐miRNA‐transfected ADSCs with or without an exosomal inhibitor.

The advancement of miRNA therapies has been impeded by the lack of an effective delivery method. To address this issue exosomes have been used to deliver exogenous mRNA and miRNA to target cells 16, 21, 22, 23. For example, TGF‐β1 mRNA has been transported by exosomes to initiate tissue repair regenerative responses and activation of fibroblasts in a mouse kidney injury model 16. Lou *et al*. 21 showed that miR‐122‐transfected AMSCs secreted miR‐122 exosomes, which could then mediate miR‐122 communication between AMSCs and hepatocellular carcinoma cells to render cancer cells more sensitive to chemotherapeutic agents. Intratumour injection of exosomes containing miR‐122 could significantly increase the anti‐tumour efficacy of sorafenib on hepatocellular carcinoma cells in a mouse model. MSC‐derived exosomes were shown to elicit hepatoprotective effects against toxicant‐induced injury, mainly through activation of proliferative and regenerative responses in a CCl4‐induced liver injury model in mice 22. By exploiting the characteristics of exosome excretion, we have shown that up‐regulated expression of fibrotic genes in HST‐T6 cells induced by TGF‐β1 was repressed following the addition of isolated exosomes containing miR181‐5p compared with exosomes containing the control miR‐67 from C. elegans. We found that this exosome therapy could attenuate liver injury and significantly down‐regulate the fibrotic components collagen I, vimentin, α‐SMA and fibronectin in the livers of mice, compared with controls. We have demonstrated that miR‐181‐5p could be delivered to target cells through exosomes in a CCl4‐induced liver fibrosis murine model and can alleviate the level of fibrosis in liver tissue.

By modifying the 3′UTR of STAT3 and Bcl‐2, we demonstrate that miR‐181‐5p attenuates STAT3 and Bcl‐2 gene expression. Stat3 cross‐linking TGF‐β1 signalling is known to play an important role in increasing fibrosis‐related products in a rat diethylinitrosamine‐induced liver fibrosis model and in HSCs 8. Furthermore, TGF‐β1 cannot achieve profibrogenic cytokine and anti‐apoptosis characteristics without Stat3 activation in HSCs. In fact, there are two Stat3‐binding sites in the promoter region of TGF‐β1, which have been demonstrated to activate TGF‐β1 in T cells 24. STAT3 has also been found to mediate the transcription of Bcl‐2 25, 26, and TGF‐β1 has been observed to increase the expression of Bcl‐2 *via* the MAPK/ERK pathway 27. Bcl‐2 under the control of miR‐449a was also found to play a role in the progression of pulmonary fibrogenesis 27. Our findings reveal that fibrogenic pathways are regulated by exosomal delivery of miR‐181‐5p in a traditional way.

We found that miR181‐5p down‐regulated STAT3 and Bcl‐2 and activated autophagy in HST‐T6 cells. Furthermore, we demonstrate that the miR‐181 down‐regulated STAT3 pathway mediated TGF‐β1 signalling to have an important effect on inflammation. Other miRNAs have also been found to activate autophagy in response to liver injury. MiR‐26a enhances autophagy in both cultured cells and the mouse liver by increasing the expression of the autophagy mediator Beclin‐1 28. Knockdown of Beclin‐1 by siRNA protected the cells from sorafenib‐induced autophagy 29. The inhibition of Mcl‐1 by sorafenib was found to disrupt the Beclin‐1‐Mcl‐1 complex, and as sorafenib did not alter levels of Beclin 1, it is possible that the role played by sorafenib is through interfering with the binding of Beclin 1 with Mcl‐1. In this work, we have discovered that, knockdown of Beclin‐1 by siRNA protected the cells from miR181‐5p‐ADSC‐induced autophagy and propose that miR‐181‐5p induces autophagy by inhibiting the STAT3/Bcl‐2/Beclin 1‐dependent pathway.

## Conclusions

We have demonstrated that exosomes containing miR‐181‐5p can increase autophagy and reduce TGF‐β1‐induced liver fibrosis by inhibiting the STAT3/Bcl‐2/Beclin 1 pathway in HST cells and a CCl4‐induced liver fibrosis mouse model. The effective anti‐fibrotic function of engineered ADSCs is able to selectively transfer miR‐181‐5p in exosomes to damaged liver cells and will lead to the development of exosomal therapeutic delivery of miRNAs targeting liver disease.

## Conflict of interest

The authors declare that they have no competing interests.

## Authors' contributions

Dr. Lungen Lu and Ying Qu designed research; Dr. Ying Qu performed research; Dr. Qidi Zhang, Xiaobo Cai, Fei Li and Zhenzeng Ma contributed new reagents/analytic tools and analysed data; and Dr Mingyi Xu and Lungen Lu wrote the manuscript.
